# Association of Tumor Size with Risk of Lymph Node Metastasis in Clear Cell Renal Cell Carcinoma: A Population-Based Study

**DOI:** 10.1155/2020/8887782

**Published:** 2020-10-31

**Authors:** Yunlai Zhi, Xiao Li, Feng Qi, Xin Hu, Wenbo Xu

**Affiliations:** ^1^Department of Urology, Lianyungang Clinical College of Nanjing Medical University & The First People's Hospital of Lianyungang, Lianyungang 222002, China; ^2^Department of Urologic Surgery, Jiangsu Cancer Hospital & Jiangsu Institute of Cancer Research & Affiliated Cancer Hospital of Nanjing Medical University, Nanjing 210009, China; ^3^First Clinical Medical College of Nanjing Medical University, Nanjing 210029, China; ^4^Department of Urology, The Fifth Affiliated Hospital of Zhengzhou University, Zhengzhou, Henan 450008, China

## Abstract

The purpose of this article was to explore the association of tumor size with lymph node metastases (LNM) risk in patients with clear cell renal cell carcinoma (ccRCC). Based on the Surveillance, Epidemiology, and End Result (SEER) database, patients diagnosed with ccRCC from 1988 to 2015 were included in this study. For each patient, personal characteristics, clinicopathological data, and survival outcomes were, respectively, collected. Subsequently, the odds ratios (ORs) and 95% confidence intervals (CIs) were calculated to investigate the potential risk factors for LNM in ccRCC. Finally, Kaplan-Meier (KM) survival plots of overall survival (OS) and ccRCC-specific survival (CSS) were evaluated on the basis of different tumor sizes. A total of 8,292 patients were finally enrolled in the study, 1,170 of whom (14.11%) had LNM. According to the heatmap, we could intuitively interpret that larger tumor size was related to an increased risk of LNM obviously. The risk of LNM was evidently greater for larger tumor size (4-7 cm: OR = 2.415, 95% CI = 1.708–3.415; 7–10 cm: OR = 3.746, 95% CI = 2.677–5.242; and >10 cm: OR = 4.617, 95% CI = 3.302–6.457) compared with smaller tumor size (≤4 cm). According to the KM survival plots of OS and CSS, we observed a gradual decline in survival with increasing tumor size, while the smallest tumor size had the best survival outcomes. These results indicated the positive relationship of tumor size with risk of LNM in ccRCC. And we also noticed continual decrease survival rates of OS and CSS with increasing tumor size.

## 1. Introduction

Kidney cancer remains a serious public health problem in the world. The estimated new cases and deaths in the United States in 2020 are 73,750 and 14,830, respectively [1]. Renal cell carcinoma (RCC) is the most common type of kidney cancer, and clear cell RCC (ccRCC) accounts for probably over 80% of all RCC cases [2].

Modern cross-sectional imaging has significantly improved the diagnosis of RCC, especially the small asymptomatic renal masses [3]. Previous study reported that approximately 30% of RCC patients developed lymph node metastases (LNM) or distant metastases at their initial diagnosis [4]. Furthermore, the 5-year survival rate of metastatic patients fell below 10%, and the median survival time rarely exceeded 1 to 2 years [4, 5]. Substantial researches have demonstrated that RCC patients with LNM may not benefit from nephrectomy and lymph node dissection (LND) [6, 7]. Although some immunosuppressants and targeted drugs have been established as first-line therapy in metastatic RCC, the majority of patients still relapsed and died within several years [8]. Kroeger et al. [9] reported that patients with LNM had worse prognosis compared to those without LNM even though they have been treated with targeted drugs previously.

Considering the high probability rate of metastasis at the time of diagnosis and extremely poor prognosis, it is urgently necessary to assess the risk of LNM for newly diagnosed RCC patients. Many studies have been performed to investigate the risk factors of LNM associated with RCC [9–12]. Additionally, the relationship of tumor stage and tumor grade with high risk of LNM in RCC has been confirmed in previous study [11]. Moreover, Rosiello et al. [12] found that histological subtype and nuclear grade were closely associated with the risk of LNM in patients receiving nephrectomy. However, to the best of our knowledge, the relationship between tumor size and LNM in ccRCC is still unknown. Tumor size plays an important role in the diagnosis and treatment of RCC, and it is a decisive factor in operative type (partial nephrectomy or not). Besides, tumor size is tightly related to the classification of T stage in RCC [13, 14].

Hence, the purpose of our research was to explore the relationship of tumor size with LNM risk in patients with ccRCC. Specifically, we first recognized and extracted eligible ccRCC patients from the Surveillance, Epidemiology, and End Results (SEER). Then the rates of LNM and Kaplan–Meier (KM) survival plots were evaluated on the basis of different tumor sizes. In consideration of that tumor size might act as an important potential risk factor for LNM, our findings could provide useful advice for clinical practice.

## 2. Methods

### 2.1. Database

All data utilized in our research were retrospectively obtained from the SEER database. As the largest population-based cancer database in the United States, SEER program (https://seer.cancer.gov) collects and publishes information of cancer patients in 18 registries, covering nearly 30% of the US population. We signed the data agreement and contacted the database with the username 15440-Nov2018. Moreover, the use of public database was exempt by the approval of Institutional Review Board (IRB).

### 2.2. Patient Identification

The original data of patients diagnosed with ccRCC were identified and collected from SEER database using the SEER∗Stat software (version 8.3.6; National Cancer Institute, Bethesda, USA). Patients enrolled in this study should fulfill the following criteria: (1) histologically confirmed as ccRCC (C74.9, International Classification of Diseases for Oncology: 8310/3) between 1988 and 2015, (2) ccRCC being the first primary malignancy for each patient, (3) complete data being available with active follow-up. Additionally, the exclusion criteria were as follows: (1) patients with bilateral tumors or the laterality being unknown, (2) age at diagnosis being younger than 18 years, (3) LND not performed or LNM status unknown, (4) patients with preoperative radiotherapy, (5) missing/ineligible data on T stage, cause of death, the administration of surgery, and so on, (6) reporting source being autopsy/death certificate only.

### 2.3. Data Extraction

We extracted the clinical characteristics and long-term follow-up outcomes by the “Case Listing Session” tool in the SEER∗Stat software, variables including age at diagnosis, race, sex, year of diagnosis, tumor laterality, T stage, tumor grade, the administration of surgery and lymph node removal, LNM status, vital status, survival months, cause of death, and so on. Based on the tumor size, all patients were categorized into four groups: “≤4 cm,” “4–7 cm,” “7–10 cm,” and “>10 cm.” In this study, age at diagnosis was considered as categorical variable with a 10-year interval. Race was classified as White, Black, other (containing American Indian/AK Native, Asian/Pacific Islander), or unknown. Tumor grade was classified into Grade I (well differentiated), Grade II (moderately differentiated), Grade III (poorly differentiated), and Grade IV (undifferentiated). Overall survival (OS) was defined as the time from ccRCC diagnosis until any cause of death or the date of the last follow-up visit. ccRCC-specific survival (CSS) was defined as the time from ccRCC diagnosis to death caused by ccRCC.

### 2.4. Statistical Analysis

All variables were calculated with descriptive statistics. Univariate and multivariate logistic regression analyses were performed to explore the association between tumor size and LNM. As mentioned, personal characteristics and clinicopathological data were analyzed using the multivariate logistic regression model to recognize potential risk factors for LNM. All the potential risk factors were included as covariates to perform an adjusted model. Additionally, KM survival analyses were constructed to estimate the impact of tumor size on OS and CSS. Differences between four groups were assessed by Log-rank test. SPSS 23.0 software (SPSS Inc., Chicago, IL, USA) and R version 3.6. 1 (http://www.r-project.org/) were used for all statistical analyses. *P* < 0.05 was considered as statistically significant (two-sided).

## 3. Results

### 3.1. Baseline Characteristics

**Figure 1:**
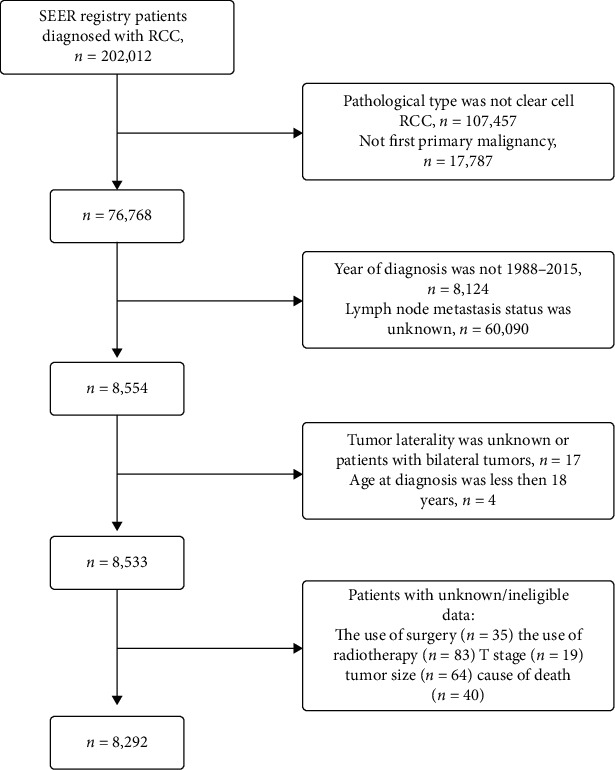
The study flow diagram of the selection process.

Patient selection process is shown in Figure 1. Ultimately, a total of 8,292 ccRCC patients were included in the study, 1,179 of whom (14.11%) had LNM. Generally, all four tumor size groups were highly representative, and the tumor size distribution was as follows: ≤4 cm: *n* = 1,212 (14.62%), 4–7 cm: *n* = 2,278 (27.47%), 7–10 cm: *n* = 2,428 (29.93%), and >10 cm: *n* = 2,320 (27.98%). Most ccRCC patients were male (65.06%) and White (85.40%). Further details about the population demographics and baseline characteristics are shown in Table 1.

### 3.2. Relationship between Tumor Size and LNM

**Figure 2:**
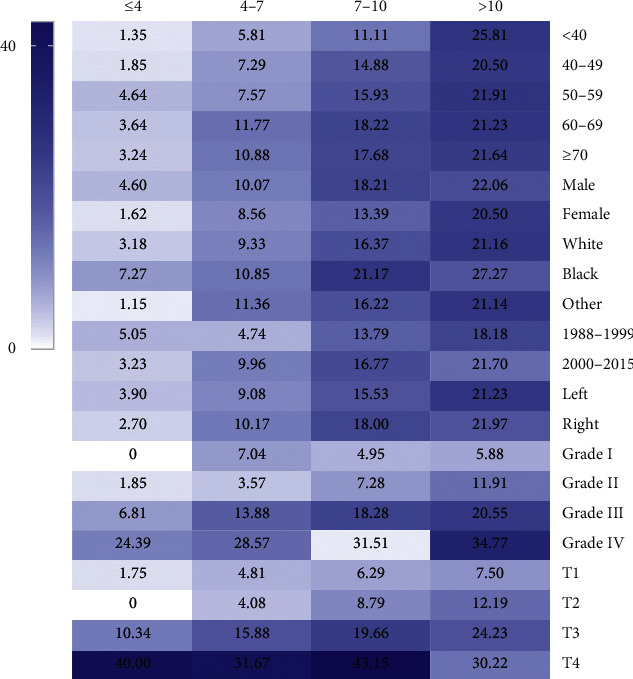
Heatmap showing rate of lymph node metastases (LNM) of clear cell renal cell carcinoma among patients with tumor size of ≤4, 4–7, 7–10, and >10 cm stratified by different characteristics, respectively. The darker the colour, the higher the risk of LNM.

Firstly, we assessed the relationship between tumor size and LNM. As shown in Table 1, larger tumor size was significantly correlated with an increased risk of LNM. The rate of LNM was lowest (3.38%) in ccRCC patients with smaller tumor size (≤4 cm), and the rate progressively increased to 21.55% in patients with tumor size larger than 10 cm. As shown in Figure 2, we could intuitively interpret that a larger tumor size was obviously related to an increased risk of LNM, regardless of the age, sex, race, year of diagnosis, tumor laterality, T stage, and tumor grade. Moreover, we also investigate the rates of LNM in ccRCC patients stratified by T stage (Table 2). Data for the table showed displayed that larger tumor size hold higher risk of LNM, except for patients with T4 diseases (*P*=0.220).

**Figure 3:**
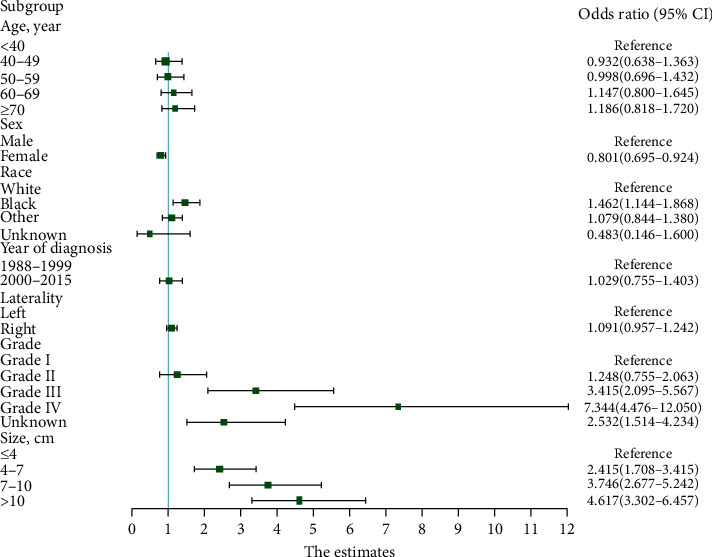
Forest plot showing results of multivariate logistic regression model for identifying potential risk factors for lymph node metastases in patients with clear cell renal cell carcinoma.

Multivariate logistic regression analysis confirmed the relationship between LNM and tumor size. Several factors were selected as covariates to perform an adjusted model including age at diagnosis, sex, race, year of diagnosis, tumor laterality, grade, T stage, and tumor size (Table 3). Results demonstrated that sex, race, grade, and tumor size (all *P* < 0.05) were identified to be risk factors for LNM in patients with ccRCC. As shown in Figure 3, the risk of LNM was evidently greater for larger tumor size (4–7 cm: OR = 2.415, 95% CI = 1.708–3.415; 7–10 cm: OR = 3.746, 95% CI = 2.677–5.242; and >10 cm: OR = 4.617, 95% CI = 3.302–6.457) compared with smaller tumor size (≤4 cm). Positive correlation (*P* < 0.001) could be detected between tumor size and LNM in the multivariate logistic model.

Moreover, we examined the association in subgroups according to T stage, sex, grade, laterality, race, year of diagnosis, and age, respectively (Supplemental Table 1). Most of odds ratios listed in factors groups mentioned above (except T2 stage, Grade I, Grade III, Grade IV, Black, diagnosed between 1988 and 1999, and age <70 years) were significant (*P* < 0.05) even after adjusting for confounders. In other words, subgroup analyses indicated a clear positive correlation between tumor size and risk of LNM.

### 3.3. Survival Outcomes

**Figure 4:**
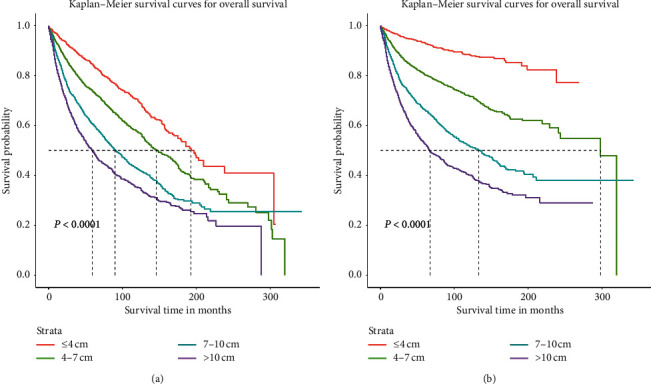
Comparison of cause-specific survival and overall survival among patients with clear cell renal cell carcinoma with tumor size of ≤4, 4–7, 7–10, and >10 cm. Cause-specific survival (a) and overall survival (b).

KM survival analyses were carried out to learn the effect of different tumor size on OS and CSS (Figure 4). According to the KM survival plots of OS (Figure 4(a)), we observed a gradual decline in survival with increasing tumor size, and the smallest tumor size holds the longest survival outcomes. For CSS, similar results were observed in Figure 4(b).

## 4. Discussion

To our knowledge, this is the first comprehensive research to explore the relationship between tumor size and the risk of LNM in patients with ccRCC. Our study indicated that tumor size was an important risk factor for LNM in ccRCC, and a larger tumor size was obviously related to an increased risk of LNM. According to the KM survival plots of OS and CSS, we noticed continual decrease in survival rates with increasing tumor size.

Previous studies found that RCC cases with smaller tumor volume had lower risk of metastasis and better prognosis [15, 16]. Herrlinger et al. demonstrated that only one case (1/740) with less than 3 cm in diameter was metastatic. In addition, larger tumor size was closely related to poorer survival outcomes in ccRCC patients [15]. A study that enrolled 2,691 metastatic RCC patients found that patients with tumors smaller than 30 mm had negligible risk of metastatic; they also found that tumor size was significantly associated with long-term prognosis [16]. Kates et al. investigated the prevalence metastatic and locally advanced RCC in the SEER database, and they concluded that patients with small renal masses (2.5–3.0 cm) also had a greater probability of metastatic potential [17]. In our study, with the increase of tumor size, the risk of metastasis also increased. Similarly, ccRCC patients with small tumor size also had a certain LNM probability, especially for those with T4 stage and Grade IV diseases. Hence, it was still necessary to pay close attention to LNM for patients with small renal masses.

In this study, sex, race, tumor grade, and tumor size were identified to be risk factors for developing LNM. In ccRCC patients, histological type, pathological grade, and clinical stage were tightly related to the survival outcomes [18–20], which were dependent on excision biopsy and histological examination. Hence, tumor size judged by clinicians plays an important role in evaluating malignancy and prognosis [21]. As for RCC, many studies showed that two cut-points of aggressiveness tumor sizes may be 4 and 7 cm [17, 22, 23]. As for RCC, many studies have explored the important role of tumor size in diagnosis and prognosis [22, 24]. Frank et al. [22] identified 2,770 adult patients who underwent radical nephrectomy or nephron sparing surgery between 1970 and 2000 to examine the relationship between tumor size and malignancy among RCC. They found that larger tumor size was tightly associated with higher malignancy. Schips et al. [24] demonstrated that RCC patients with tumor larger than 5 cm were more likely to develop symptoms when compared with those with smaller tumors. Meanwhile, those with suspicious symptoms had a 1.8-fold greater risk of dying of cancer when compared with those without symptoms. In our study, we found that patients with the smallest tumor size held the longest survival outcomes. Therefore, tumor size provides important information in assessing the severity of the disease and the long-term prognosis. Moreover, this also reminds us of the importance of finding small lesions in regular physical examinations to achieve early detection, early diagnosis, and early treatment in clinical work.

Interestingly, our study found that sex and T stage were risk factors for developing LNM in ccRCC patients. Compared with male patients, female patients had lower risk of LNM. Kates et al. found that male patients were 1.51 times more likely to develop LNM than females [17]. This may be attributed to suppression of RCC growth via estrogen/estrogen receptor signaling pathway [25]. Additionally, we found that higher T stage was closely associated with higher risk of LNM. More aggressive RCC in high T stage might promote LNM, which should be further confirmed in future studies.

Several shortcomings of our research should be noted. First of all, previous study has reported that tumor size of RCC from SEER database (1998–2003) had certain error rate, but the subsequent tumor sizes had been amended [26]. As a result of a long-time span of our study (1988–2015), the influence as narrated above may be limited. Secondly, some variables were missing in the SEER registry which may limit further analysis of this study. Third, it was a retrospective study based on a public database. Accordingly, our results need to be confirmed in prospective studies. Although the SEER registry has its unavoidable limitations, it is one of the best cancer databases due to the large sample size.

## 5. Conclusion

Our research revealed that tumor size is an important risk factor for LNM in ccRCC. And we also noticed continual decrease of survival rates of OS and CSS with increasing tumor size. These findings could provide useful advice for clinical practice.

## Figures and Tables

**Table 1:** Baseline characteristics of enrolled patients (*n* = 8,292), stratified by tumor size.

	Total	≤4 cm	4–7 cm	7–10 cm	>10 cm
*N*	8,292 (100)	1,212 (14.62)	2,278 (27.47)	2,482 (29.93)	2,320 (27.98)
Age, year					
<40	352 (4.25)	74 (6.11)	86 (3.78)	99 (3.99)	93 (4.01)
40–49	1,347 (16.24)	216 (17.28)	329 (14.44)	363 (14.63)	439 (18.92)
50–59	2,520 (30.39)	345 (28.47)	634 (27.83)	797 (32.11)	744 (32.07)
60–69	2,434 (29.35)	330 (27.23)	705 (30.95)	697 (28.08)	702 (30.26)
≥70	1,639 (19.77)	247 (20.38)	524 (23.00)	526 (21.19)	342 (14.74)
Sex					
Male	5,395 (65.06)	718 (59.24)	1,460 (64.09)	1,653 (66.60)	1,564 (67.41)
Female	2,897 (34.94)	494 (40.76)	818 (35.91)	829 (33.40)	756 (32.59)
Race					
White	7,081 (85.40)	1,006 (83.00)	1,962 (86.13)	2,138 (86.14)	1,975 (85.13)
Black	541 (6.52)	110 (9.08)	129 (5.66)	137 (5.52)	165 (7.11)
Other	623 (7.51)	87 (7.18)	176 (7.73)	185 (7.45)	175 (7.54)
Unknown	47 (0.57)	9 (0.74)	11 (0.48)	22 (0.89)	5 (0.22)
Year of diagnosis					
1988–1999	533 (6.43)	99 (8.17)	190 (8.34)	145 (5.84)	99 (4.27)
2000–2015	7,759 (93.57)	1,113 (91.83)	2,088 (91.66)	2,337 (94.16)	2,221 (95.73)
Laterality					
Left	4,746 (57.42)	693 (57.18)	1,344 (59.00)	1,404 (56.57)	1,305 (56.25)
Right	3,546 (42.76)	519 (42.82)	934 (41.00)	1,078 (43.43)	1,015 (43.75)
Grade					
Grade I	459 (5.54)	165 (13.61)	142 (6.23)	101 (4.07)	51 (2.20)
Grade II	2,763 (33.32)	596 (49.17)	924 (40.56)	714 (28.77)	529 (22.80)
Grade III	2,833 (34.17)	235 (19.39)	735 (32.27)	919 (37.03)	944 (40.69)
Grade IV	1,223 (14.75)	41 (3.38)	189 (8.30)	438 (17.65)	555 (23.92)
Unknown	1,014 (12.23)	175 (14.44)	288 (12.64)	310 (12.49)	241 (10.39)
T stage					
T1	2,498 (30.13)	970 (80.03)	1,289 (56.58)	159 (6.41)	80 (3.45)
T2	1,487 (17.93)	53 (4.37)	98 (4.30)	819 (33.00)	517 (22.28)
T3	3,861 (46.56)	174 (14.36)	831 (36.48)	1,358 (54.71)	1,498 (64.57)
T4	446 (5.38)	15 (1.24)	60 (2.63)	146 (5.88)	225 (9.70)
LNM					
No	7,122 (85.89)	1,171 (96.62)	2,061 (90.47)	2,070 (83.40)	1,820 (78.45)
Yes	1,170 (14.11)	41 (3.38)	217 (9.53)	412 (16.60)	500 (21.55)

Data were *n* (%). LNM = lymph node metastases; Grade I = well differentiated; Grade II = moderately differentiated; Grade III = poorly differentiated; Grade IV = undifferentiated.

**Table 2:** Risk of LNM in ccRCC patients with different tumor sizes, stratified by T stage.

Tumor size (cm)	T1		T2		T3		T4	
	*n*	LNM rate	*n*	LNM rate	*n*	LNM rate	*n*	LNM rate
≤4	970	17 (1.75%)	53	0 (0.00%)	174	18 (10.34%)	15	6 (40.00%)
4–7	1,289	62 (4.81%)	98	4 (4.08%)	831	132 (15.88%)	60	19 (31.67%)
7–10	159	10 (6.29%)	819	72 (8.79%)	1,358	267 (19.66%)	146	63 (43.15%)
>10	80	6 (7.50%)	517	63 (12.19%)	1,498	363 (24.23%)	225	68 (30.22%)
*P* value	<0.001		<0.001		<0.001		0.220	

LNM = lymph node metastases; ccRCC = clear cell renal cell carcinoma.

**Table 3:** Multivariate logistic regression model for distinguishing potential risk factors for LNM in patients with ccRCC.

	OR	95% CI	*P*
Age, year			0.121
<40	Reference		
40–49	0.932	0.638–1.363	0.717
50–59	0.998	0.696–1.432	0.992
60–69	1.147	0.800–1.645	0.455
≥70	1.186	0.818–1.720	0.368
Sex			**0.002**
Male	Reference		
Female	0.801	0.695–0.924	0.002
Race			**0.012**
White	Reference		
Black	1.462	1.144–1.868	0.002
Other	1.079	0.844–1.380	0.543
Unknown	0.483	0.146–1.600	0.234
Year of diagnosis			0.854
1988–1999	Reference		
2000–2015	1.029	0.755–1.403	0.854
Laterality			0.192
Left	Reference		
Right	1.091	0.957–1.242	0.192
Grade			**<0.001**
Grade I	Reference		
Grade II	1.248	0.755–2.063	0.388
Grade III	3.415	2.095–5.567	<0.001
Grade IV	7.344	4.476–12.050	<0.001
Unknown	2.532	1.514–4.234	<0.001
Size (cm)			**<0.001**
≤4	Reference		
4–7	2.415	1.708–3.415	<0.001
7–10	3.746	2.677–5.242	<0.001
>10	4.617	3.302–6.457	<0.001

OR = odds ratio; CI = confidence interval; LNM = lymph node metastases; ccRCC = clear cell renal cell carcinoma; Grade I = well differentiated; Grade II = moderately differentiated; Grade III = poorly differentiated; Grade IV = undifferentiated.

## Data Availability

This study was conducted using data from NCI's SEER program of the United States (http://seer.cancer.gov/) and guided by a data use agreement between NCI and RTI Health Solutions.

## Supplementary Materials

Supplementary Materials Relationship between tumor size and lymph node metastasis after adjusting other potential risk factors for lymph node metastasis stratified by different characteristics.
