# Supplementary material for: Association of Tumor Size with Risk of Lymph Node Metastasis in Clear Cell Renal Cell Carcinoma: A Population-Based Study
Source: J Oncol. 2020 Oct 31;2020:8887782. doi: 10.1155/2020/8887782 (PMC7648693; doi:10.1155/2020/8887782)
Supplement: Supplementary Materials — Relationship between tumor size and lymph node metastasis after adjusting other potential risk factors for lymph node metastasis stratified by different characteristics. [file 8887782.f1.docx]

Supplemental Table 1: Relationship between tumor size and lymph node metastasis after adjusting other potential risk factors for lymph node metastasis stratified by different characteristics

| Subgroups | OR (95% CI) | *P* |
| --- | --- | --- |
| T1 stage |  | 0.001 |
| ≤4 cm | Reference |  |
| 4-7 cm | 2.445 (1.398-4.277) | 0.002 |
| 7-10 cm | 3.860 (1.676-8.890) | 0.002 |
| >10 cm | 4.637 (1.701-12.644) | 0.003 |
| T2 stage |  | 0.176 |
| ≤4 cm | Reference |  |
| 4-7 cm | ∞ (0,+∞) | 0.997 |
| 7-10 cm | ∞ (0,+∞) | 0.997 |
| >10 cm | ∞ (0,+∞) | 0.997 |
| T3 stage |  | 0.002 |
| ≤4 cm | Reference |  |
| 4-7 cm | 1.601 (0.940-2.726) | 0.083 |
| 7-10 cm | 1.805 (1.077-3.026) | 0.025 |
| >10 cm | 2.203 (1.318-3.683) | 0.003 |
| T4 stage |  | 0.023 |
| ≤4 cm | Reference |  |
| 4-7 cm | 1.027 (0.303-3.485) | 0.966 |
| 7-10 cm | 1.316 (0.424-4.087) | 0.635 |
| >10 cm | 0.638 (0.206-1.971) | 0.434 |
| Male |  | 0.001 |
| ≤4 cm | Reference |  |
| 4-7 cm | 1.602 (1.068-2.402) | 0.023 |
| 7-10 cm | 2.119 (1.405-3.195) | 0.000 |
| >10 cm | 2.212 (1.467-3.336) | 0.000 |
| Female |  | 0.003 |
| ≤4 cm | Reference |  |
| 4-7 cm | 3.384 (1.577-7.262) | 0.002 |
| 7-10 cm | 3.172 (1.444-6.969) | 0.004 |
| >10 cm | 4.080 (1.863-8.938) | 0.000 |
| Grade 1 |  | 0.956 |
| ≤4 cm | Reference |  |
| 4-7 cm | ∞ (0,+∞) | 0.995 |
| 7-10 cm | ∞ (0,+∞) | 0.995 |
| >10 cm | ∞ (0,+∞) | 0.995 |
| Grade 2 |  | 0.001 |
| ≤4 cm | Reference |  |
| 4-7 cm | 1.394 (0.679-2.861) | 0.365 |
| 7-10 cm | 1.939 (0.897-4.190) | 0.092 |
| >10 cm | 3.267 (1.517-7.037) | 0.002 |
| Grade 3 |  | 0.200 |
| ≤4 cm | Reference |  |
| 4-7 cm | 1.775 (1.002-3.145) | 0.049 |
| 7-10 cm | 1.876 (1.046-3.364) | 0.035 |
| >10 cm | 1.862 (1.042-3.329) | 0.036 |
| Grade 4 |  | 0.771 |
| ≤4 cm | Reference |  |
| 4-7 cm | 1.251 (0.562-2.781) | 0.583 |
| 7-10 cm | 1,272 (0.587-2.759) | 0.542 |
| >10 cm | 1.401 (0.650-3.020) | 0.389 |
| Left |  | 0.022 |
| ≤4 cm | Reference |  |
| 4-7 cm | 1.590 (1.015-2.490) | 0.043 |
| 7-10 cm | 1.657 (1.051-2.613) | 0.030 |
| >10 cm | 1.965 (1.248-3.095) | 0.004 |
| Right |  | 0.000 |
| ≤4 cm | Reference |  |
| 4-7 cm | 2.753 (1.529-4.958) | 0.001 |
| 7-10 cm | 3.842 (2.101-7.026) | 0.000 |
| >10 cm | 3.977 (2.173-7.279) | 0.000 |
| White |  | 0.000 |
| ≤4 cm | Reference |  |
| 4-7 cm | 1.967 (1.321-2.929) | 0.001 |
| 7-10 cm | 2.306 (1.540-3.455) | 0.000 |
| >10 cm | 2.646 (1.767-3.964) | 0.0.00 |
| Black |  | 0.235 |
| ≤4 cm | Reference |  |
| 4-7 cm | 1.129 (0.420-3.032) | 0.810 |
| 7-10 cm | 2.457 (0.816-7.396) | 0.110 |
| >10 cm | 2.390 (0.809-7.060) | 0.115 |
| Other |  | 0.249 |
| ≤4 cm | Reference |  |
| 4-7 cm | 7.048 (0.892-55.697) | 0.064 |
| 7-10 cm | 4.716 (0.576-38.642) | 0.148 |
| >10 cm | 5.332 (0.655-43.434) | 0.118 |
| 1988-1999 |  | 0.143 |
| ≤4 cm | Reference |  |
| 4-7 cm | 0.732 (0.220-2.440) | 0.612 |
| 7-10 cm | 1.767 (0.572-5.461) | 0.323 |
| >10 cm | 1.999 (0.619-6.457) | 0.247 |
| 2000-2015 |  | 0.000 |
| ≤4 cm | Reference |  |
| 4-7 cm | 2.083 (1.430-3.034) | 0.000 |
| 7-10 cm | 2.367 (1.608-3.485) | 0.000 |
| >10 cm | 2.644 (1.798-3.889) | 0.000 |
| Age< 40 years old |  | 0.142 |
| ≤4 cm | Reference |  |
| 4-7 cm | 2.150 (0.226-20.443) | 0.505 |
| 7-10 cm | 3.218 (0.334-31.041) | 0.312 |
| >10 cm | 6.151 (0.674 56.087) | 0.107 |
| 40-49 years old |  | 0.079 |
| ≤4 cm | Reference |  |
| 4-7 cm | 2.321 (0.767-7.022) | 0.136 |
| 7-10 cm | 3.723 (1.208-11.474) | 0.022 |
| >10 cm | 3.638 (1.191-11.110) | 0.023 |
| 50-59 years old |  | 0.124 |
| ≤4 cm | Reference |  |
| 4-7 cm | 1.139 (0.615-2.112) | 0.679 |
| 7-10 cm | 1.424 (0.768-2.639) | 0.262 |
| >10 cm | 1.711 (0.925-3.164) | 0.087 |
| 60-69 years old |  | 0.055 |
| ≤4 cm | Reference |  |
| 4-7 cm | 2.419 (1.263-4.633) | 0.008 |
| 7-10 cm | 2.314 (1.186-4.515) | 0.014 |
| >10 cm | 2.459 (1.264-4.783) | 0.008 |
| ≥70 years old |  | 0.024 |
| ≤4 cm | Reference |  |
| 4-7 cm | 2.547 (1.170-5.548) | 0.019 |
| 7-10 cm | 3.223 (1.445-7.186) | 0.004 |
| >10 cm | 3.454 (1.526-7.821) | 0.003 |

OR: odds ratio; CI: confidence interval; Grade I=Well differentiated; Grade II=Moderately differentiated; Grade III=Poorly differentiated; Grade IV=Undifferentiated.
